# Exosomal MicroRNA-320a Derived From Mesenchymal Stem Cells Regulates Rheumatoid Arthritis Fibroblast-Like Synoviocyte Activation by Suppressing CXCL9 Expression

**DOI:** 10.3389/fphys.2020.00441

**Published:** 2020-05-26

**Authors:** Qing Meng, Bing Qiu

**Affiliations:** Department of Orthopedics, Guizhou Orthopedics Hospital, Guiyang, China

**Keywords:** rheumatoid arthritis, mesenchymal stem cells, exosomes, microRNA-320a, CXCL9, fibroblast-like synoviocytes

## Abstract

Rheumatoid arthritis (RA), a chronic systemic inflammatory disease, is a primary cause of disability worldwide. The involvement of fibroblast-like synoviocytes (FLSs) in the regulation of the pathogenesis of RA has been highlighted. Mesenchymal stem cells (MSCs) are important candidates for cell-based treatment in many inflammatory autoimmune diseases. Herein, we identify whether MSC-derived exosomes loaded with microRNA-320a (miR-320a) regulate RA-FLSs. Synovial tissues from 22 patients with RA and 9 patients with osteoarthritis were collected. RA-FLSs were obtained from patients with RA, and their functions were evaluated by determining levels of interleukin-1β (IL-1β), IL-6, and IL-8 and by transwell migration and invasion assays. Dual luciferase reporter gene assays were employed to identify interaction between miR-320a and CXC chemokine ligand 9 (CXCL9). A co-culture system of MSC-derived exosomes and RA-FLSs were performed. The collagen-induced arthritis (CIA) mouse models with arthritis and bone damage were developed. Our results revealed the existence of reciprocal expression of miR-320a and CXCL9 in the synovial tissues obtained from patients with RA. CXCL9 knockdown or miR-320a upregulation suppressed the activation, migration, and invasion of RA-FLSs. CXCL9 was confirmed to be a target of miR-320a, and CXCL9 overexpression restored RA-FLS function in the presence of miR-320a. MSC-derived exosomes containing miR-320a mimic significantly suppressed RA-FLS activation, migration, and invasion *in vitro* and attenuated arthritis and bone damage in mice with CIA *in vivo*. Our study uncovers that MSC-derived exosomes participate in the intercellular transfer of miR-320a and subsequently inhibit the progression of RA. These results provide a novel potential therapeutic approach for RA treatment by increasing miR-320a in exosomes.

## Introduction

As a systemic and chronic autoimmune disease, rheumatoid arthritis (RA) influences the lining of the synovial joints often leading to disability, premature death, and social and economic burdens of human beings (Guo et al., 2018). Owing to direct disease-related and treatment-related effects, the risk of infection in patients with RA increases (Yamanaka et al., 2017). Fibroblast-like synoviocytes (FLSs) have been widely documented to be effectors of cartilage destruction in RA and play a crucial role in initiating and maintaining the inflammatory and destructive processes in the rheumatoid joint (Bottini and Firestein, 2013; Bustamante et al., 2017). Mesenchymal stem cells (MSCs) possess the ability to form bone and cartilage and also are involved in immunosuppression, so they have been considered as a new therapeutic target for RA (Sonomoto et al., 2014; Calabrese et al., 2017). Furthermore, it has been known that human MSCs have capacities of producing a mass of exosomes (Yeo et al., 2013). MSC-derived exosomes have been shown to suppress T and B lymphocytes to exert therapeutic effects on inflammatory arthritis (Cosenza et al., 2018).

Exosomes, 30- to 150-nm extracellular vesicles released by cells, have emerged as a novel endogenous delivery system (Holliday et al., 2017). The use of exosomes as biological vehicles for microRNA (miRNA) transfer represents a growing area of interest as exosomes do not pose a threat from a tumor formation perspective or lead to acute immune rejection (Hu et al., 2012). Belonging to small, non-coding RNAs, miRNAs have been reported to degrade mRNAs and repress translation by post-transcriptionally mediating gene expression (Singh et al., 2013). Previous evidence has suggested that miRNAs serve crucial regulatory roles in relation to FLS in RA and may be used as diagnostic biomarkers and therapeutic agents for RA (Hong et al., 2018). It is reported that evidence has been presented indicating that miR-320a is significantly reduced in cancer tissues and might act as a regulator of genes in certain diseases, such as prostate cancer (Okato et al., 2016), hepatocellular carcinoma (Yao et al., 2012), and myasthenia gravis (Cheng et al., 2013). Furthermore, miR-320a is downregulated in RA, inhibiting proliferation and enhancing the apoptosis of FLSs by suppressing MAPK-ERK1/2 signaling pathway (Lin et al., 2019). According to the miRNA and TargetScan databases, it was predicted that CXC chemokine ligand 9 (CXCL9) was one of the target genes of miR-320a. High CXCL9 expression has been identified in the synovial tissues of patients with RA, which participates in the pathogenesis and inflammatory process in RA (Kotrych et al., 2015). The above information led to a hypothesis that MSC-derived exosomal miR-320a might be correlated to RA with involvement of CXCL9. Therefore, the present study was conducted with the central objective of providing theoretical support for the treatment of RA by elucidating the mechanism of exosomal miR-320a from MSCs in the activation, migration, and invasion of RA-FLS.

## Materials and Methods

### Ethics Statement

Informed consent agreement forms were signed and collected from all participants prior to enrollment into our study. Guizhou Orthopedics Hospital provided ethical approval for this study, which also conformed to the *Declaration of Helsinki*. Animal experiments were conducted in strict accordance with the Guide to the Management and Use of Laboratory Animals issued by the National Institutes of Health. The protocol of animal experiments was approved by the Institutional Animal Care and Use Committee of Guizhou Orthopedics Hospital.

### Rheumatoid Arthritis and Osteoarthritis Sample Collection

The specimens of this study were obtained from patients in the Department of Rheumatism and Immunology of Guizhou Orthopedics Hospital from October 2015 to October 2018. The synovial tissues, taken from arthroplasty or synovectomy, were from 22 patients with RA (six males and 16 females, average age: 38.59 years) and 9 patients with osteoarthritis (OA) (three males and six females, average age: 45.22 years) (Lee et al., 2017). All patients fulfilled the diagnosis of the American College of Rheumatology for RA and OA (Yu et al., 2019). The synovial tissues were divided into two parts: one part was used for separation of RA-FLSs and molecular detection, and the other part was routinely embedded in paraffin and then cut into 4-μm sections for the use of immunohistochemistry and hematoxylin and eosin (H&E) staining.

### Rheumatoid Arthritis Fibroblast-Like Synoviocyte Isolation and Identification

The synovial tissues were collected, cut under sterile conditions, detached with 10 mg/ml of type II collagenase (Gibco BRL, Gaithersburg, MD, United States) at 37°C for 4 h and centrifuged. The cells were then collected and cultured in Dulbecco’s modified Eagle’s medium (DMEM) (HyClone Laboratories, Inc., Logan, UT, United States) containing 10% fetal bovine serum (FBS; 10099141, Gibco BRL). Cell passage was conducted every 3 days, followed by 24-h adherent growth of cells at 37°C with 5% CO_2_. Next, phosphate-buffered saline (PBS) was utilized to remove the suspended cells. The cells were detached with trypsin-ethylenediaminetetraacetic acid and passaged after reached 85% confluence. The RA-FLSs at passage 3 to 5 were utilized for the following experiments (Ye et al., 2018).

### Isolation and Identification of Bone Marrow Mesenchymal Stem Cells

Bone marrow specimens of MSCs were provided by three patients (two males and one female, aged from 26 to 52 years) with femoral head necrosis from the Guizhou Orthopedics Hospital. The diagnosis was based on the results of magnetic resonance imaging. Those patients had no loss of femoral head height and without diseases such as trauma, blood system, binding, and tumor infiltration. MSCs were isolated from three donor bone marrows according to the predecessor method (Bing et al., 2012) and cultured in DMEM-F12 containing 10% FBS, 0.2% bi-anti-penicillin, and streptomycin (HyClone Laboratories). The passage was performed every 3 days, and MSCs at passage 3 to 7 were used for subsequent experiments. The MSCs were then cultured in OriCell MSC osteolyzed, adipogenic, or cartilage-differentiated medium (Cyagen Biosciences Inc., Guangzhou, China) on the basis of the manufacturer’s instructions, followed by Alizarin Red staining, oil red O staining, and Alcian blue staining for the identification of osteogenic differentiation, adipogenic differentiation, and chondrogenic differentiation, respectively (Dong et al., 2018).

### Flow Cytometry

RA-FLSs and MSCs were collected and washed twice with PBS and stained with antibodies purchased from Abcam (Cambridge, United Kingdom): fluorescein isothiocyanate (FITC)-labeled human leukocyte antigen (HLA)-G (ab239334, 1:500), HLA-DR (ab1182, 1 μg/10^6^ cells), CD4 (ab59474, 10 μg/10^6^ cells), CD14 (ab28061, 10 μg/10^6^ cells), CD19 (ab24936, 1 μg/10^6^ cells), CD34 (ab18227, 20 μl/100 μl of sample), CD44 (ab25064, 10 μg/10^6^ cells), CD45 (ab27287, 20 μg/10^6^ cells), CD55 (ab180646, 1:50), CD73 (ab239246, 4 μg/10^6^ cells), CD90 (ab226, 10 μg/10^6^ cells), and CD105 (ab53318, 20 μg/10^6^ cells); then they were thoroughly mixed with PBS solution containing 1% horse serum keeping for 1 h and fixed with 80% alcohol. The same concentration of goat anti-mouse immunoglobulin G (IgG) isotope antibody conjugated with FITC was then added as a negative control (NC; 1:1,000, BD Biosciences Pharmingen, San Jose, CA, United States). The fluorescence-activated cell sorting (FACS) Verse instrument (BD Biosciences Pharmingen) was employed in order to evaluate the test samples, with the results analyzed using FlowJo software (Tree Star, Ashland, OR, United States).

### Isolation of Exosomes

Fetal bovine serum was centrifuged at 100,000 × *g* for 18 h to remove the exosomes in the serum. When MSC confluence reached approximately 80%, the supernatant of the culture medium was removed, followed by two PBS washes. MSCs were continually cultured in 10% exosome-depleted FBS at 37°C with CO_2_ for 48 h. The culture supernatant of MSCs at logarithmic phase was collected for isolation of exosomes. Initially, the cells and medium mixture were centrifuged for 10 min at 500 × *g*, with the precipitants subsequently removed. The supernatant was collected and the centrifugation was repeated once. Next, the supernatant was aspirated and centrifuged for 30 min at 2,000 × *g* to remove cell debris. The supernatant was filtered through a 220-nm filter and centrifuged at 100,000 × *g* for 90 min. The supernatant was discarded, and the exosomes subsided as a pellet, which was collected. Next, the pellet was resuspended in sterile PBS and centrifuged for 60 min at 100,000 × *g* at 4°C, and the supernatant was removed to eliminate protein contamination. After washing, suspending, and precipitating again, the precipitate was resuspended in PBS, followed by filtration and sterilization with 0.22-nm filter and frozen at −20°C for reserve (Thery et al., 2006; Epple et al., 2012). Exosome markers CD9 (ab92726, 1:2,000, Abcam) and CD63 (ab118307, 1:50, Abcam) and endoplasmic reticulum marker calnexin (ab75801, 1:1,000, Abcam) were used to identify the exosomes by Western blot analysis.

### Transmission Electron Microscope

The exosome resuspension was ultracentrifuged, and precipitate was obtained and fixed with fixative (2% paraformaldehyde and 2.5% glutaraldehyde) at 4°C for 1 h. The precipitate was washed with PBS three times (15 min/time), fixed with 1% citric acid for 1.5 h, and washed with PBS three times (15 min/time). Afterward, the precipitate was dehydrated using graded ethanol and then saturated with epoxy resin overnight, embedded, and polymerized at 35°C, 45°C, and 60°C for 24 h, followed by observation of radiography under a transmission electron microscope (TEM) (Delong LVEM5, Delong America, Montreal, QC, Canada) after ultrathin sectioning and uranyl staining.

### Nanoparticle Tracking Analysis

The exosome resuspension was serially diluted and detected in a nanoparticle tracking analyzer (Malvern Instruments Ltd., Malvern, Worcestershire, United Kingdom) at a concentration of (1–9) × 10^8^ cells/ml. The appropriate background gray scale was selected in the operating software to record the movement trajectory of the particles, and the concentration and particle size distribution map of the diluted sample were output. The concentration of exosomes in the original solution was calculated by dilution multiple.

### Western Blot Analysis

The cells were lysed using radioimmunoprecipitation assay lysis buffer (Solarbio Science and Technology Corporation, Beijing, China). The protein concentration was estimated using a bicinchoninic acid protein assay kit. Next, 50 μg of protein was dissolved in 2 × sodium dodecyl sulfate (SDS) loading buffer and boiled at 100°C. After 5 min, the protein was separated by 10% SDS–polyacrylamide gel electrophoresis and transferred onto polyvinylidene fluoride membrane. The membrane was blocked with 5% skimmed milk powder at ambient temperature for 1 h and incubated at 4°C overnight with the following primary rabbit antibodies purchased from Abcam: CXCL9 (ab9720, 0.3 μg/ml) and glyceraldehyde-3-phosphate dehydrogenase (GAPDH) (ab181602, 1:10,000). The membrane was rinsed with Tris-buffered saline and Tween 20 (TBST) for three times, subsequently incubated with horseradish peroxidase-labeled secondary IgG antibody (1:1,000) for 1 h, and rinsed with TBST three times. The membrane was placed on a clean glass plate, developed with enhanced chemiluminescence kit (BB-3501, Amersham Pharmacia Biotech, Little Chalfont, Bucks, United Kingdom), and photographed by IS gel image analysis system (Alpha Technologies Services Inc., Hudson, OH, United States). The results were analyzed using ImageJ software.

### Determination of Uptake of Exosomes

The exosomes were labeled with PKH26 fluorescent dye (Sigma Aldrich, St Louis, MO, United States): 200 μg of exosomes and 4 μl of PKH26 fluorescent dye were added into 1 ml of Diluent C solution (Beyotime Biotechnology, Shanghai, China), after which the aforementioned two mixtures were mixed gently for 5 min. After centrifugation at 100,000 × *g* (4°C) for 2 h, the supernatant was discarded, with the mixture then washed twice with PBS and finally centrifuged at 100,000 × *g* (4°C) for 2 h followed by collection of the labeled exosomes. RA-FLSs were plated in a 24-well plate (5 × 10^4^ cells/well), followed by overnight culture. A total of 100 μg of labeled exosome solution was added to the medium, with an equal amount of PBS solution as the blank control. In 5% CO_2_ incubator at 37°C, a Nikon Eclipse Ti confocal laser scanning microscopy (Nikon, Tokyo, Japan) was utilized to observe the uptake of exosomes by RA-FLSs at 24, 48, and 72 h; and the impact of exosomes on the activation, metastasis, and invasion of RA-FLSs was further analyzed.

### Cell Transfection

The 70% confluent RA-FLSs were seeded into a 6-well plate containing serum-free DMEM. Mimic-NC, miR-320a mimic, small interfering RNA (si)-NC, si-CXCL9-1, and si-CXCL9-2 plasmids (Guangzhou RiboBio Co., Ltd., Guangzhou, China) were then transiently transfected into RA-FLSs using Lipofectamine 2000 (Invitrogen, Carlsbad, CA, United States). After 24 h, the RA-FLSs were harvested for subsequent experiments.

The mimic-NC and miR-320a mimic plasmids were transfected into MSCs with the same method as above. The exosomes were isolated from transfected MSCs (Exo-NC and Exo-miR-320a) 24 h later.

### Activation Detection of Rheumatoid Arthritis Fibroblast-Like Synoviocytes

The RA-FLSs at the logarithmic growth phase after transfection were seeded into a 35-mm culture dish containing serum-free medium at a density of 1 × 10^5^ cells/well for 24 h. After starvation, the RA-FLSs were incubated with 10 ng/ml of tumor necrosis factor-α (TNF-α) for 10 min, and then the levels of interleukin-1β (IL-1β), IL-6, and IL-8 in the culture medium were measured by enzyme-linked immunosorbent assay (ELISA) according to the instructions of the kits (BOSTER Biological Technology Co., Ltd., Wuhan, Hubei, China) (Lao et al., 2019).

### Enzyme-Linked Immunosorbent Assay

A total of 10 g of synovial tissues was taken to prepare tissue homogenate, and 3 g of homogenate was used. The level of CXCL9 in synovial tissue was detected using a CXCL9 ELISA kit (BOSTER). The levels of CXCL9, IL-1β, IL-6, and IL-8 in RA-FLS supernatant and mouse serum were directly detected using an ELISA kit (BOSTER). All the aforementioned operations were performed according to the instructions of the respective kits.

### Reverse Transcription Quantitative Polymerase Chain Reaction

Total RNA was extracted from cells using TRIzol (16096020; Thermo Fisher Scientific Inc., Waltham, MA, United States). A total of 5 μg of RNA was reversely transcribed into complementary DNA (cDNA) using cDNA Kit (K1622; Fermentas Inc., Ontario, CA, United States). The expression of miR-320a was determined using TaqMan miRNA assay (Ambion, Austin, TX, United States) with U6 employed as the loading control. The expression of CXCL9 was determined using PrimeScript RT-PCR kit (TaKaRa, Shiga, Japan), with GAPDH utilized as the loading control. The primers used are shown in Table 1. The gene expression fold changes between the experiment group and the control group was calculated based on the 2^–ΔΔ*Ct*^ method.

**TABLE 1:** Primer sequences for RT-qPCR.

Genes	Primer sequences (5′-3′)
MiR-320a	F: CCTGGTGTAAACTCCTCGCTG
	R: AACTGTGTCGTGTAGTCG
U6	F: GCTTCGGCAGCACATATACTAAAAT
	R: CGCTTCAGAATTTGCGTGTCAT
CXCL9	F: GGAGTGCAAGGAACCCCAGTA
	R: CTTTTGGCTGACCTGTTTCTC
GAPDH	F: GTTAGTGGGGTCTCGCTCTG
	R: CAGGACGCGCAAACATGA

*F, forward; R, reverse; RT-qPCR, reverse transcription quantitative polymerase chain reaction; miR-320a, microRNA-320a; CXCL9, CXC chemokine ligand 9; GAPDH, glyceraldehyde-3-phosphate dehydrogenase.*

### Dual Luciferase Reporter Gene Assay

The artificially synthesized 3′ untranslated region (3′UTR) gene fragment of CXCL9 was constructed into pMIR-reporter (Beijing Huayueyang Biotechnology Co., Ltd., Beijing, China) using endonuclease *Spe*I and *Hin*dIII. Next, a mutation site of the complementary sequence of the seed sequence on the CXCL9 wild type (WT) was designed, and corresponding plasmid was constructed. The luciferase reporter plasmids WT and mutation (MUT) were co-transfected with mimic-NC, miR-320a mimic, inhibitor-NC, and miR-320a inhibitor plasmids into HEK-293T cells (CRL-1415, Shanghai Xinyu Biological Technology Co., Ltd., Shanghai, China). Luciferase activity was measured using a luciferase assay kit (RG005, Beyotime Biotechnology, Shanghai, China) using a Glomax 20/20 luminometer (Promega, Madison, WI, United States). The results are presented as the activity of the Renilla luciferase (M2)/the activity of firefly luciferase (M1).

### Transwell Assay

#### Migration

The cells at logarithmic growth phase were resuspended in serum-free DMEM, and the cell density was adjusted to 3 × 10^5^ cells/ml. A total of 100 μl of cell suspension was added into the apical chamber of a 24-well transwell chamber, and 500 μl of DMEM containing 10% FBS was pre-added into basolateral chamber. The chamber was placed in a 37°C incubator with 5% CO_2_ for 24 h, then washed with PBS, fixed with methanol for 10 min, and stained with the crystal violet for 10 min. The cells on the upper chamber were carefully wiped using a cotton swab, and the chamber membrane was obtained. In total, six randomly selected fields of the chamber membrane were observed under a microscope of 200 times for counting.

#### Invasion

Fifty microliters of Matrigel (BD Biosciences Pharmingen) was pre-filled in the apical chamber of the transwell chamber and air-dried for 4 h at ambient temperature, and the other procedures were the same as migration.

### Establishment and Identification of Collagen-Induced Arthritis Model

Healthy and clean C57BL/6 male mice [aged 7 weeks and weighing 21.35 g, the Experimental Animal Center of Guizhou Medical University (certificate number: 0278832), Guizhou, China] were randomly equally separated into two groups. Among them, 13 normal mice did not receive any treatment as control, and another 13 mice were utilized for establishing collagen-induced arthritis (CIA) model. Then, 2 mg/ml of Chondrex chicken type II collagen (C II, Chondrex, Redmond, WA, United States) was mixed with equal volume of 5 mg/ml of Freund’s complete adjuvant to prepare a type II collagen emulsion. Then 0.1 ml of type II collagen emulsion was intradermally injected into the back and tail of mice. On the 21st day post basic immunization, the injection was repeated to perform booster immunization by avoiding the primary immunization site. Control mice were injected with an equal volume of normal saline as described above.

After 21 days of booster immunization, the arthritis index (AI) was used as a criterion for estimating whether the model was successfully established. Swelling grade of limb joints was scored on a scale of 0–IV: 0, no redness; I, slightly swollen toe joint; II, swelling of the toe joint and foot joint; III, swelling of the foot below the ankle joint; and IV, all joints including the ankle joint were swollen. The AI value was the sum of four limb joint swelling grade scores (0 level, 0 point; 1 level, 1 point; total score, 16 points). The mouse was regarded as a successful model if its AI value ≥ 4.

The mice were then euthanized, the hind legs of the diseased mice were cut along the roots, and the excess tissues were removed. The metatarsophalangeal joints and interphalangeal joints were preserved, washed with PBS, and decalcified with 8% nitric acid solution for 16–18 h. The joints were embedded in paraffin and sectioned at 5 μm, followed by H&E staining. During H&E staining, the arthritis score (0–4 points) was estimated as follows: 0 point, normal joints; 1 point, mild focal infiltration; 2 points, moderate infiltration; 3 points, severe inflammatory infiltration without angiogenesis or cartilage damage; 4 points, a large number of angiogenesis and severe invasion in articular cartilage and subchondral bone (Gu et al., 2014).

C57BL/6 male mice were initially utilized to establish the CIA model on the basis of the aforementioned methods, after which they were separated into three groups. On the second day of the booster immunization of CIA mice, PBS, Exo-miR-NC, and Exo-miR-320a were injected into the tail vein of the mice at a dose of 100 μg/day (*n* = 13/treatment) (Zhang et al., 2019) until 1 day prior to the completion of the experiment. After 21 days of booster immunization, the mice were anesthetized, and the eyeballs were removed to collect blood. The serum levels of CXCL9, IL-1β, IL-6, and IL-8 were measured by ELISA. After the mice were euthanized, the metatarsophalangeal joint and the interphalangeal joint were taken, followed by H&E staining and immunohistochemistry. Finally, the inflammatory score was assessed.

### Hematoxylin and Eosin Staining

The operation was performed according to the reference (Li et al., 2016).

### Immunohistochemistry

The procedure was performed in accordance with the instructions of the streptavidin–biotin complex immunohistochemical staining Kit (ZSGB-Bio Technology Co., Ltd., Beijing, China). Primary rabbit antibodies of CXCL9 (ab9720, 0.3 μg/ml), IL-1β (ab2105, 1:100), IL-6 (ab208113, 1:50), and IL-8 (ab106350, 1 μg/ml) and secondary goat anti-rabbit IgG antibody (ab150077, 1:500) were used (Juczynska et al., 2017).

### Statistical Analysis

Statistical analysis was conducted using SPSS 21.0 statistical software (IBM Corp. Armonk, NY, United States). Measurement data were expressed as mean ± standard deviation. Data of two groups were compared by independent sample *t* test. Data among multiple groups were analyzed by one-way analysis of variance (ANOVA), followed by Tukey’s *post hoc* test. The correlation between miR-320a and CXCL9 expression was analyzed by Pearson’s correlation analysis. Difference in *p* value of <0.05 was statistically significant.

## Results

### Silence of CXCL9 Inhibited the Activation, Migration, and Invasion of Rheumatoid Arthritis Fibroblast-Like Synoviocytes

**FIGURE 1:**
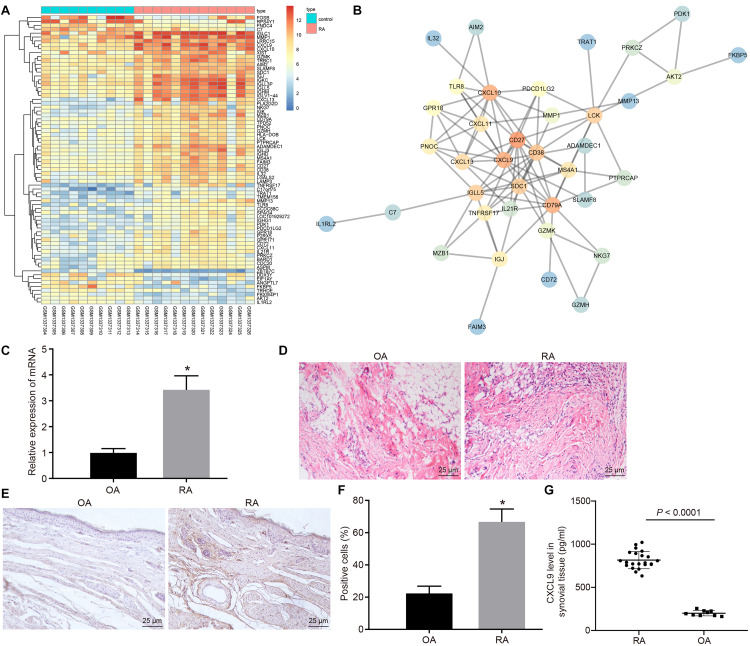
CXC chemokine ligand 9 (CXCL9) was highly expressed in synovial tissues of patients with rheumatoid arthritis (RA). Osteoarthritis (OA) patients, *n* = 9; RA patients, *n* = 22. **(A)** Differential expressed genes (74 genes) in normal samples and disease samples analyzed in RA-related GSE55457 microarray data of Gene Expression Omnibus (GEO) database. **(B)** Interaction analysis of differentially expressed genes. **(C)** H&E staining of the synovial pathological features of patients with OA and RA (×400). **(D)** RT-qPCR detection of CXCL9 mRNA expression in synovial tissues of patients with OA and RA. **(E)** Immunohistochemistry of CXCL9 positive protein expression in the synovial tissues of patients with OA and RA (×400). **(F)** Statistical analysis of panel **(E)**. **(G)** The level of CXCL9 in synovial tissues of patients with OA and RA detected by ELISA method. Statistical analysis was performed using an independent sample *t* test. Data were expressed as mean ± standard deviation. *p < 0.05 compared with OA patients.

Gene expression in normal and disease samples in the RA-related GSE55457 microarray data from the Gene Expression Omnibus (GEO) database was analyzed to obtain 74 differentially expressed genes (Figure 1A). The interaction analysis of the differentially expressed genes was performed in order to construct a gene interaction network. The results revealed that CD27, CD79A, CXCL10, and CXCL9 were at the center of the entire network (Figure 1B). Previous study has found that CXCL9 is associated with RA (Kuan et al., 2010), but the mechanism of CXCL9 in RA has not been clarified. Therefore, we focused on the regulatory mechanism of CXCL9 in RA. Initially, the synovial tissues of patients with RA and OA were collected. The results of H&E staining revealed that in comparison with that of patients with OA, the inflammatory cell infiltration was observed in the synovial tissues of patients with RA, and the angiogenesis was significantly increased (Figure 1C). The results of reverse transcription quantitative polymerase chain reaction (RT-qPCR) revealed that CXCL9 was highly expressed in the synovial tissues of patients with RA in comparison with patients with OA (*p* < 0.05; Figure 1D). Immunohistochemistry and ELISA also showed high expression of CXCL9 in the synovial tissues of patients with RA (*p* < 0.05; Figures 1E–G).

**FIGURE 2:**
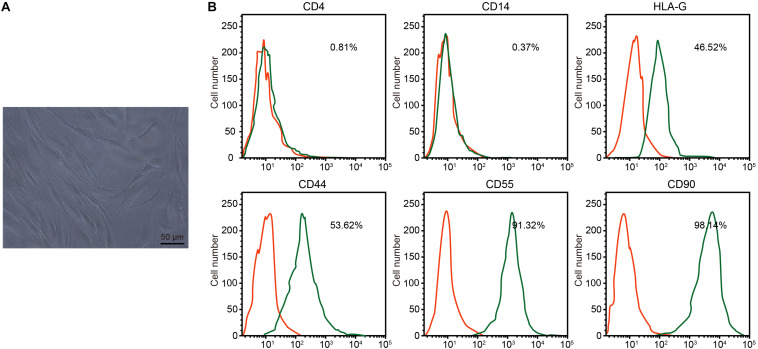
Isolation and identification of rheumatoid arthritis fibroblast-like synoviocytes (RA-FLSs). **(A)** RA-FLS morphology observed under an inverted microscope (×200). **(B)** Expression of RA-FLS surface markers detected by flow cytometry.

Under the microscope, RA-FLSs exhibited typical spindle-shaped morphology and vortical growth pattern (Figure 2A). Next, the RA-FLS surface markers were detected by flow cytometry. The isolated cells had low CD4 and CD14 signals, medium HLA-G and CD44 signals, and strong CD55 and CD90 signals, confirming that those cells were RA-FLSs (Figure 2B).

**FIGURE 3:**
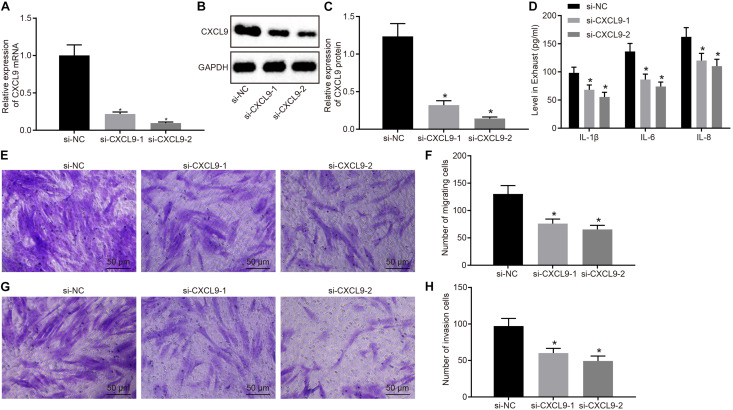
Silencing of CXC chemokine ligand 9 (CXCL9) repressed the activation, migration, and invasion of rheumatoid arthritis fibroblast-like synoviocytes (RA-FLSs). RA-FLSs were treated with si-CXCL9-1, si-CXCL9-2, or si-NC. **(A)** CXCL9 mRNA expression in RA-FLSs detected by RT-qPCR. **(B,C)** Western blot analysis of protein expression of CXCL9 in RA-FLSs normalized to GAPDH. **(D)** The levels of IL-1β, IL-6, and IL-8 in RA-FLSs detected by ELISA assay. **(E,F)** RA-FLS migration ability (×200) detected by transwell assay. **(G,H)** RA-FLS invasive ability (×200) detected by transwell assay. Data were expressed as mean ± standard deviation and analyzed by one-way ANOVA, followed by Tukey’s *post hoc* test. *p < 0.05 compared with RA-FLSs treated with si-NC. Cell experiment was repeated three times independently.

Next, RA-FLSs were treated with si-CXCL9-1, si-CXCL9-2, or si-NC to explore the role of CXCL9 in activation, migration, and invasion of RA-FLSs. CXCL9 expression was significantly decreased by treatment of si-CXCL9-1 or si-CXCL9-2 (Figures 3A–C). RA-FLSs were stimulated with 10 ng/ml of TNF-α to activate cells and then treated with si-NC, si-CXCL9-1, or si-CXCL9-2. Then ELISA was conducted, which showed that IL-1β, IL-8, and IL-6 levels were decreased after CXCL9 was inhibited (Figure 3D). Transwell assay illustrated that the use of si-CXCL9-1 or si-CXCL9-2 significantly reduced migration and invasion of RA-FLSs (Figures 3E–H). The above results demonstrated that CXCL9 was highly expressed in patients with RA and that CXCL9 silencing could inhibit the activation, migration, and invasion of RA-FLSs.

### MiR-320a Targetedly Inhibited CXCL9

**FIGURE 4:**
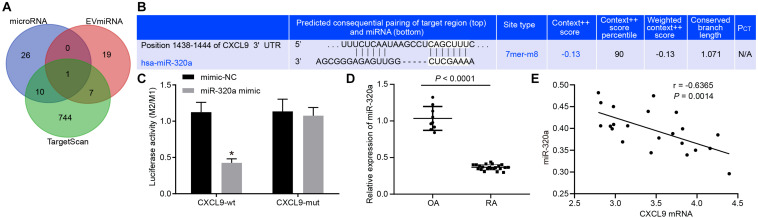
CXC chemokine ligand 9 (CXCL9) was targeted by miR-320a. **(A)** miRNAs in mesenchymal stem cell (MSC)-derived exosomes predicted in EVmiRNA database. **(B)** Upstream regulatory miRNAs of CXCL9 predicted in microRNA database and TargetScan database. **(C)** Luciferase activity of CXCL9-WT and CXCL9-MUT detected by dual luciferase reporter gene assay; the experiment was repeated three times independently. **(D)** MiR-320a mRNA level in synovial tissue of patients with osteoarthritis (OA) (*n* = 9) and patients with rheumatoid arthritis (RA) (*n* = 22) detected by RT-qPCR. **(E)** Pearson’s correlation analysis of expression of miR-320a and CXCL9 in synovial tissues of 22 patients with RA. Statistical analysis was performed using an independent sample *t* test. Data were expressed as mean ± standard deviation. *p < 0.05 compared with the treatment of mimic-NC group or OA patients.

The miRNAs in MSC-derived exosomes were searched in EVmiRNA database, and miRNAs with expression values above 10 were selected for subsequent analysis. At the same time, the upstream regulatory miRNAs of CXCL9 were predicted by miRNA database and TargetScan database, and intersected with results in EVmiRNA database, which exhibited that the intersection gene was miR-320a (Figures 4A,B). The luciferase reporter gene assay results showed that the luciferase activity of CXCL9-WT was significantly decreased by miR-320a mimic (Figure 4C). RT-qPCR revealed that miR-320a was poorly expressed in patients with RA (Figure 4D). Then, correlation between miR-320a and CXCL9 expression was analyzed by Pearson’s correlation analysis. As depicted in Figure 4E, miR-320a expression was negatively correlated with CXCL9 expression (*r* = −5.517, *p* < 0.05). Based on the above results, CXCL9 was a target gene of miR-320a.

### Overexpressed MiR-320a Suppressed the Activation, Migration, and Invasion of Rheumatoid Arthritis Fibroblast-Like Synoviocytes by Downregulating CXCL9

**FIGURE 5:**
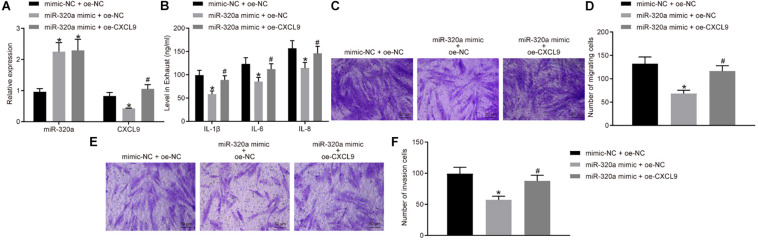
Upregulation of miR-320a repressed the activation, migration, and invasion of rheumatoid arthritis fibroblast-like synoviocytes (RA-FLSs) *via* CXC chemokine ligand 9 (CXCL9). RA-FLSs were treated with mimic-NC + oe-NC, miR-320a mimic + oe-NC, or miR-320a mimic + oe-CXCL9. **(A)** RT-qPCR detection of miR-320a expression and CXCL9 mRNA expression in RA-FLSs. **(B)** The levels of IL-1β, IL-6, and IL-8 in RA-FLSs detected by ELISA. **(C,D)** The migration ability of RA-FLSs (×200) detected by transwell assay. **(E,F)** The invasive ability of RA-FLSs (×200) detected by transwell assay. Statistical analysis was performed using one-way ANOVA, followed by Tukey’s *post hoc* test. Data were expressed as mean ± standard deviation. *p < 0.05 compared with RA-FLSs treated with mimic-NC + oe-NC; #*p* < 0.05 compared with RA-FLSs treated with miR-320a mimic + oe-NC. Cell experiment was repeated three times independently.

In order to further investigate the effects of miR-320a on RA-FLS activation, migration, and invasion, RA-FLSs were transfected with miR-320a mimic and oe-CXCL9. The miR-320a expression was significantly increased, and CXCL9 expression was decreased after treatment of miR-320a mimic + oe-NC compared with the treatment of mimic-NC + oe-NC, whereas CXCL9 expression was increased after transfected with miR-320a mimic + oe-CXCL9 (all *p* < 0.05; Figure 5A). The results of ELISA and transwell assay showed that the activation, migration, and invasion of RA-FLSs were decreased following treatment of miR-320a mimic + oe-NC, which was rescued by treatment of miR-320a mimic + oe-CXCL9 (Figures 5B–F). In conclusion, miR-320a upregulation inhibited the activation, migration, and invasion of RA-FLSs, and overexpressed CXCL9 reversed the effect of miR-320a on RA-FLSs.

### MiR-320a Was Highly Enriched in Exosomes Derived From Mesenchymal Stem Cells

**FIGURE 6:**
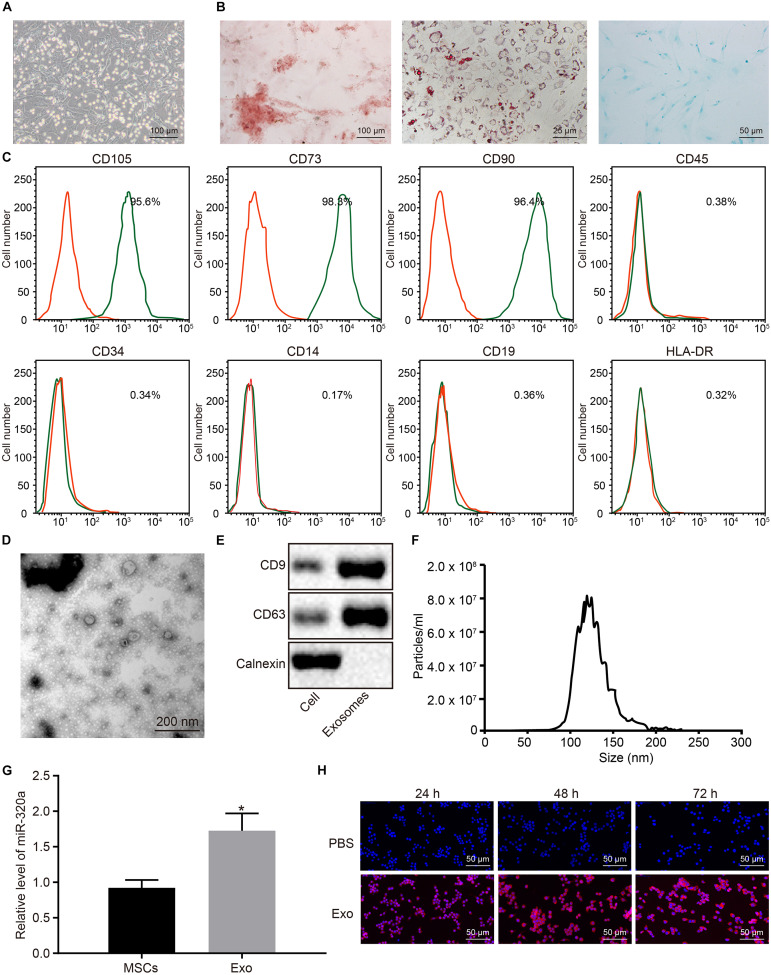
MiR-320a was highly enriched in exosomes from mesenchymal stem cells (MSCs). **(A)** The morphology of the fifth passage of MSCs observed by the inverted microscope (×100). **(B)** The formation of osteoblast, adipocytes, and chondrocytes analyzed by cytochemical staining performed by Alizarin Red (I, ×100), oil red O (II, ×400), and Alcian blue (III, ×200), respectively. **(C)** The expression of surface marker proteins associated with MSCs determined by flow cytometry. **(D)** Observation of the ultrastructure of exosomes by transmission electron microscope (TEM) (×5,000). **(E)** The expression of CD9 and CD63 determined by Western blot analysis. **(F)** The exosome concentration and particle size by nanoparticle tracking analysis (NTA). **(G)** The expression of miR-320a in rheumatoid arthritis fibroblast-like synoviocytes (RA-FLSs) after co-culture with exosomes measured by RT-qPCR. **(H)** The uptake of MSC-derived exosomes by RA-FLSs (×200) observed by laser confocal microscopy. Statistical analysis was performed using an independent sample *t* test. Data were expressed as mean ± standard deviation. *p < 0.05 compared with MSCs or RA-FLSs cultured with PBS.

Next, in order to elucidate the effects of MSC-derived exosomal miR-320a on RA, MSCs were isolated from three donors’ bone marrows. The isolated MSCs grew in fibrous aggregation under the microscope (Figure 6A). After differentiation induction, the MSCs were differentiated into osteoblasts, adipocytes, and chondrocytes (Figure 6B). MSCs were subsequently identified using flow cytometry by evaluating the MSC markers. As illustrated in Figure 6C, CD105, CD73, and CD90 proteins were positively expressed in MSCs, whereas CD45, CD34, CD14, CD19, and HLA-DR were negatively expressed. The exosomes were then extracted from MSCs and identified. Under the TEM, the exosomes derived from MSCs were observed to have circular and elliptical membranous vesicle disk-like structures with a diameter of approximately 40–100 nm (Figure 6D). Additionally, Western blot analysis results suggested that MSC-derived exosomes expressed the marker proteins CD9 and CD63 and did not express calnexin (Figure 6E). Nanoparticle tracking analysis (NTA) revealed that the main particle size of vesicles ranged between 30 and 150 nm, which was in accordance with microscope results (Figure 6F). The results of RT-qPCR showed that miR-320a was upregulated in MSC-derived exosomes (Figure 6G). Under the laser confocal microscopy, no red fluorescence in RA-FLSs cultured with PBS was detected. Numerous red vesicles were identified to have gradually infiltrated into the RA-FLSs when the RA-FLSs were cultured with MSC-derived exosomes, with the majority of them found to be concentrated in the cytoplasm. The signal of PKH26 increased over time, indicating that exosomal uptake was highly efficient (Figure 6H). To sum up, miR-320a was highly expressed in MSC-secreted exosomes, and exosomes were absorbed by RA-FLSs.

### Mesenchymal Stem Cell-Derived Exosomal MiR-320a Targeted CXCL9 and Inhibited Activation, Migration, and Invasion of Rheumatoid Arthritis Fibroblast-Like Synoviocytes

**FIGURE 7:**
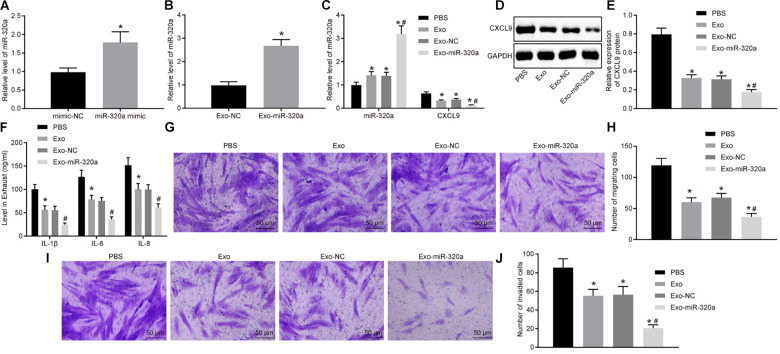
Overexpressed MSC-secreted exosomal miR-320a inhibited CXC chemokine ligand 9 (CXCL9) expression and activation, migration, and invasion in rheumatoid arthritis fibroblast-like synoviocytes (RA-FLSs). **(A)** miR-320a expression in MSCs treated with miR-320a mimic detected by RT-qPCR. **(B)** miR-320a and CXCL9 expression in exosomes isolated from MSCs treated with miR-320a mimic detected by RT-qPCR. **(C)** miR-320a and CXCL9 expression in RA-FLSs cultured with MSC-derived exosomes detected by RT-qPCR. **(D,E)** The protein level of CXCL9 in RA-FLSs cultured with MSC-derived exosomes normalized to GAPDH detected by Western blot analysis. **(F)** The levels of IL-1β, IL-6, and IL-8 in RA-FLSs cultured with MSC-derived exosomes detected by ELISA. **(G,H)** Cell migration ability of RA-FLSs cultured with MSC-derived exosomes detected by transwell assay (200×). **(I,J)** Cell invasive ability of RA-FLSs cultured with MSC-derived exosomes (200×) detected by transwell assay. Statistical analysis was performed using independent sample *t*-test or one-way ANOVA with Tukey’s *post hoc* test. Data were expressed as mean ± standard deviation. *p < 0.05 compared with RA-FLSs cultured with phosphate-buffered saline (PBS), and #*p* < 0.05 compared with RA-FLSs cultured with Exo-NC.

In order to further investigate the function of exosomal miR-320a from MSCs, the exosomes were extracted from MSCs treated with miR-320a mimic (Figure 7A). Next, RT-qPCR was employed to detect the miR-320a expression in the isolated exosomes. The expression of miR-320a was obviously enhanced in Exo-miR-320a (Figure 7B). The RA-FLSs were cultured with the above isolated exosomes, and miR-320a and CXCL9 expression in RA-FLSs was detected by RT-qPCR and Western blot analysis. The results showed that the expression of miR-320a was apparently elevated and CXCL9 was decreased in RA-FLSs by co-culture with MSC-derived exosomes or Exo-miR-320a (Figures 7C–E). Further studies showed that the activation, migration, and invasion of RA-FLSs were obviously downregulated by co-culture with MSC-derived exosomes or Exo-miR-320a (Figures 7F–J). The above results suggested that exosomes delivered miR-320a into RA-FLS, thereby targeting CXCL9 and inhibiting the activation, migration, and invasion of RA-FLSs.

### Mesenchymal Stem Cell-Secreted Exosomal MiR-320a Inhibited the Expression of Immune Factors and the Severity of Arthritis in Collagen-Induced Arthritis Mice

**FIGURE 8:**
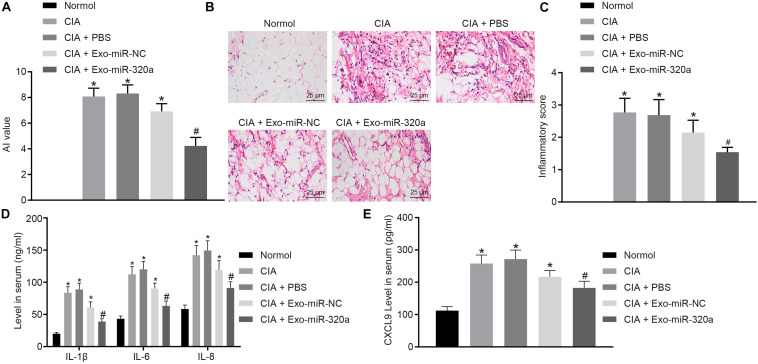
Mesenchymal stem cell (MSC)-derived exosomal miR-320a decreased the expression of immune factors and the severity of arthritis in collagen-induced arthritis (CIA) mice. CIA mice were injected with phosphate-buffered saline (PBS), Exo-miR-NC, or Exo-miR-320a. **(A)** Evaluation of arthritis index (AI) values of CIA mice. **(B,C)** H&E staining of pathological features of CIA mice (×400). **(D)** The levels of IL-1β, IL-6, and IL-8 measured by ELISA. **(E)** CXC chemokine ligand 9 (CXCL9) level in CIA mice measured by ELISA. Statistical analysis was performed using one-way ANOVA, followed by Tukey’s *post hoc* test. Data were expressed as mean ± standard deviation. *p < 0.05 compared with normal mice, and #*p* < 0.05 compared with CIA mice injected with Exo-miR-NC.

Collagen-induced arthritis model was then established to explore the effect of MSC-derived exosomal miR-320a on RA *in vivo*. The results displayed that CIA mice has obvious inflammatory reaction, severe joint deformity, and increased AI value, suggesting that all model mice were successfully established. AI value of mice was significantly reduced by injection with Exo-miR-320a (Figure 8A). H&E staining results showed that CIA mice had a large number of inflammatory cell infiltration, synovial tissue hyperplasia, partial articular cartilage destruction, and significant increased inflammatory score. After mice were injected with Exo-miR-320a, inflammatory cell infiltration, the number of synovial cells, and inflammatory score decreased obviously (Figures 8B,C). The above results demonstrated that the CIA model mice were successfully induced and that the tail vein injection of Exo-miR-320a could significantly alleviate the inflammatory symptoms of CIA mice. The results of ELISA showed that the levels of CXCL9, IL-1β, IL-6, and IL-8 in the serum of CIA mice were high. Following injection with Exo-miR-320a, the levels of CXCL9, IL-1β, IL-6, and IL-8 in serum of CIA mice were significantly decreased (Figures 8D,E). Collectively, miR-320a that secreted by MSC-derived exosomes reduced the expression of immune factors and alleviated the severity of arthritis in CIA mice.

## Discussion

Rheumatoid arthritis triggers accumulation of inflammation that has a primary effect on joints (Nevius et al., 2016). RA-FLS represents a vital mediator known to modulate the local immune microenvironment (Yang et al., 2017). Exosomes are nanometer-sized vesicles released from a wide variety of cell types capable of performing as coordinators of intercellular communication by means of transferring protein and RNA. More specifically, certain exogenous miRNAs have been shown to be packaged into exosomes and delivered into cells (O’Loughlin et al., 2012; Zhang et al., 2015). A previous study demonstrated that MSCs are suited for the extensive production of exosomes and show immunosuppressive effects (Yeo et al., 2013). The current study demonstrated a novel tactic for inhibiting the activation, migration, and invasion of RA-FLSs through miR-320a that was transferred by exosomes from MSCs. In conclusion, miR-320a-stimulated MSCs effectively packaged miR-320a into secreted exosomes, inducing communication between MSCs and RA-FLSs, thus further inhibiting the activation, migration, and invasion of RA-FLSs through downregulation of CXCL9.

Initially, our experimental data portrayed that synovial tissues of patients with RA contained high CXCL9 expression and that following knockdown of CXCL9, the activation, migration, and invasion of RA-FLSs were decreased. Previous evidence demonstrated that CXCL9, a member of the CXC chemokine family, is significantly upregulated in patients with RA (Kuan et al., 2010), which was in line with our study. The serum level of CXCL9 is strongly linked with inflammation in patients with RA (Kotrych et al., 2015). The ectopic expression of CXCL9 enhances migration, proliferation, and invasion of oral cavity squamous cell carcinoma cells and liver cancer cells *in vitro* (Chang et al., 2013; Lan et al., 2014).

Next, the results obtained from bioinformatics prediction in combination with dual-luciferase reporter gene assay indicated that miR-320a was able to bind to the 3′UTR of CXCL9, demonstrating that CXCL9 was a target gene of miR-320a. It has been reported that miR-320a is poorly expressed in patients with myasthenia gravis and regulates the inflammatory cytokines production through targeting mitogen-activated protein kinase 1 (Cheng et al., 2013). miR-320a expression is decreased in synovial tissues of patients with RA and can repress proliferation and enhance apoptosis of FLSs by suppressing the MAPK-ERK1/2 signaling pathway (Lin et al., 2019). Previous studies have highlighted the low miR-320a expression in human nasopharyngeal carcinoma, tongue squamous cell carcinoma, and salivary adenoid cystic carcinoma, whereas restored expression of miR-320a can reduce nasopharyngeal carcinoma cell migration and invasion (Qi et al., 2014; Sun et al., 2015; Xie et al., 2016), which demonstrate the role of miR-320a in regulating cellular biological processes in various diseases.

Subsequently, another critical finding was that MSC-derived exosomes containing miR-320a decreased expression of immune factors and the severity of arthritis in CIA mice. MSCs are effective on anti-inflammation and immune regulation, facilitating RA treatment (Ansboro et al., 2017). MSC-derived exosomes exert anti-inflammatory effects on inflammatory arthritis (Cosenza et al., 2018). It is authenticated that MSCs are able to potentially moderate neurite outgrowth by transferring exosomal miR-133b to neural cells (Xin et al., 2012). Further investigation has revealed that exosome-delivered miRNAs have a regulatory effect on the immune regulation and can act as the therapeutic target for autoimmune diseases including RA (Bullerdiek and Flor, 2012). Furthermore, miR-320a in exosomes derived from cancer-associated fibroblasts can suppress cell proliferation, migration, and metastasis in hepatocellular carcinoma through competitively binding to its target gene PBX3 (Zhang et al., 2017).

## Conclusion

**FIGURE 9:**
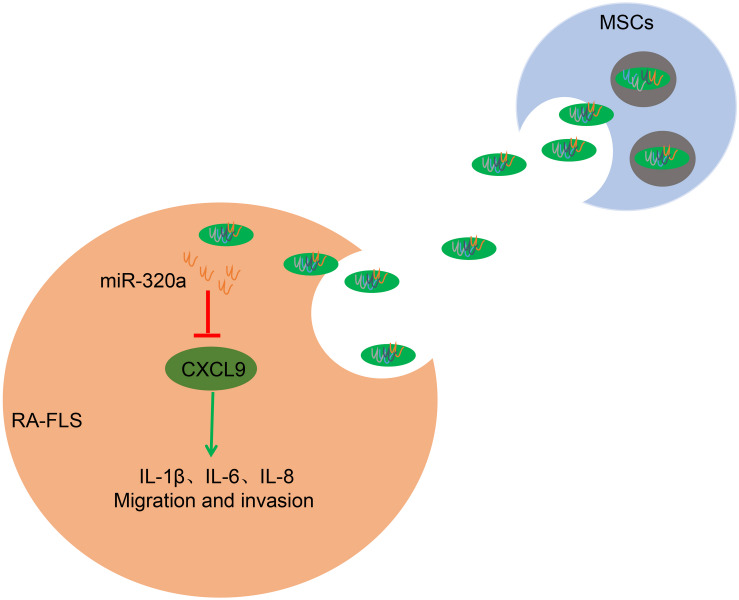
The mechanism of mesenchymal stem cell (MSC)-released exosomal miR-320a on RA *via* CXC chemokine ligand 9 (CXCL9). Highly enriched miR-320a in MSCs exosomes could be endocytosed by rheumatoid arthritis fibroblast-like synoviocytes (RA-FLSs). Endocytic exosomes released miR-320a in RA-FLSs and miR-320a can targetedly inhibit CXCL9, which activates RA-FLSs and promotes IL-1β, IL-6, IL-8 expression, and RA-FLS migration and invasion.

In conclusion, the current study demonstrated that the overexpression of miR-320a in exosomes derived from MSCs downregulated CXCL9, inhibiting the activation, migration, and invasion of RA-FLSs and repressing the expression of immune factors and the severity of RA in CIA model mice (Figure 9). These findings insinuated that miR-320a might pose as a promising diagnostic biomarker and therapeutic target in RA. At present, the effects of miR-320/CXCL9 in RA remain scantly, and we will further discuss the underlying rules that govern their interaction in our further work. What is more, taking many factors such as reagents and methods into account, the isolation of exosomes and the culture of MSCs at present are immature for clinical use, whereas more advanced and effective instruments for MSC culture and exosome purification could help to increase the probability and security of MSC-derived exosome therapy from a practical application perspective.

## Data Availability Statement

The datasets generated during the current study are available.

## Ethics Statement

Informed consent agreement forms were signed and collected from all participants prior to enrollment into our study. Guizhou Orthopedics Hospital provided ethical approval for this study, which also conformed to the *Declaration of Helsinki*. Animal experiments were conducted in strict accordance with the Guide to the Management and Use of Laboratory Animals issued by the National Institutes of Health. The protocol of animal experiments was approved by the Institutional Animal Care and Use Committee of Guizhou Orthopedics Hospital.

## Author Contributions

QM and BQ designed the study. QM collated the data, carried out data analyses, and produced the initial draft of the manuscript. BQ contributed to drafting the manuscript. Both authors have read and approved the final submitted manuscript.

## Conflict of Interest

The authors declare that the research was conducted in the absence of any commercial or financial relationships that could be construed as a potential conflict of interest.
